# The Physiology of Fighting

**Published:** 1896-01-18

**Authors:** 


### The Hospital

An Institutional Journal of

### The Medical Sciences and Hospital Administration, with

AN EXTRA NURSING SUPPLEMENT.

Vol. XIX.—No. 486.] [Jan. 18, 1896.

### THE PHYSIOLOGY OF FIGHTING.

Fighting means brain, nerve, physique, food. When done on a small scale it means all these; when carried out on an imperial scale still more does it mean the brain and nerve, which are impossible without food and physique. Mr. Gladstone tried to set the civilised world some object lessons in peaceful methods of settling international quarrels. The civilised world has shown nothing but contempt for Mr. Gladstone's object lessons. It blustered long ago; it blusters still; and its blustering not seldom leads to conflicts, appalling in their deadliness. At the present moment it is as plain as the sun at noon that there are possibilities of warfare before the modern world. We Britons have certain unwritten, but well understood, rules about declaring war against other nations. Shakespeare, three centuries ago, gave us our cue, and we have instinctively acted ever since upon his sententious utterance of our national convictions. "Beware of entrance to a quarrel, but being in, bear 't that the opposed may beware of thee." Thus our great dramatist; thus also our own convictions and actions.

Science tells us that if we are to do things as well as they can be done, we must understand scientifically all the necessary conditions of the doing of them. The most indispensable of all the conditions of successful fighting is, not weapons, but men. The most indispensable of all the endowments of fighting men are morale and physique. To produce men of the highest possible morale and physique is the business of physiology.

The doctor keeps the gateways of entrance to the army and navy in this country. He can, therefore, exercise some check upon the introduction of men of poor physique. But the doctor is obliged to work with such material as is supplied to him, and if men of the best physique are not sent for examination, then, inasmuch as a certain number of additional men must be admitted every year, he must pass the second best, or even the third best, and so on. Now, as "no man can make a silk purse out of a sow's ear," so no doctor can make stout-hearted and tough-bodied soldiers out of second, third, or fourth rate recruits.

There is then a question antecedent to that of the medical examination of recruits, and even to that of recruiting. It is the question of offering to all classes of men in this country such conditions of military service and such rewards for conduct and efficiency as will tempt men of the highest morale and physique to enter upon and to continue in a military career. This is not a question of tempting men into the ranks merely, but of inducing men of education, the most capable we have in brain and body, to choose the military career in preference to the Church, the law, medicine, or the higher walks of commerce. And here, again, comparative physiology comes to the aid of the scientific statesman by showing him that all through the animal world, from the highest mammal to the lowest coelenterate, those creatures which are best endowed with offensive and defensive weapons, and above all with the courage and skill to use them, succeed best in that struggle for existence which is as universal, as unintermitting, and as remorseless as the march of the sea upon the shore. The struggle for existence among nations, we are firmly persuaded, will never cease until nations themselves are all submerged in that universal cataclysm which prophets and even men of science so often predict. If the struggle for existence among nations be really so inevitable, and so ceaseless in duration, then it is certain that those national organisms which are best endowed with weapons of offence and defence, and with the highest skill and determination in using them, will not only survive, but prevail. On the other hand, those which have not weapons, or worse still, which have them but lack the courage and determination to use them to the very last extremity, will fail, sink behind and become extinct, like the extinct animals, great as well as small, which have disappeared in the "survival" struggles of past ages.

All these are commonplaces among biologists; they are becoming, or have become, proved conclusions among the students of social evolution. What is now insisted upon is that they must become practical, working, every-day convictions among statesmen and all the constituent elements of the State. To induce young men of the best muscle and physique, and young men of all ranks, to adopt the military career is the physiological mandate of the hour for all modern peoples. To make of such men, not merely drilled machines, but thinking brains, brave hearts, and agile and fatigue-enduring bodies is the business of these physiologists which wise statesmen will tempt to seek a career in the defence and development of the State.

In short, science must be brought into our military activities. Not the science merely of weapons, nor even of military tactics; bnt the science which makes masculine, bold, invincible men. It is here that the voice of physiology asks to be heard. Men of the right stamp must be persuaded to come to you, she says to the military authorities. When they have come you must select them with judgment. When they are selected you must instruct and develop their brains; you must make them hopeful and ambitious by offering them adequate rewards for efficiency and good service; you must feed them liberally and well; you must clothe them comfortably and smartly so that they may respect themselves; you must so provide for them that they shall feel they may risk death fearlessly, knowing that those dependent upon them will be cared for by the State. Cromwell's Ironsides constituted almost the finest fighting machine the world has ever seen; and that because every man was himself a possible Cromwell at heart. If physiological statesmanship could have its way it would provide Great Britain, if that were possible, with a million of men like Cromwell's Ironsides, and respect and pay them as such men would deserve to be respected and paid. Magnificent is the future which lies before the scientific nations. Is Britain wise enough and great enough in science and in manhood to take her place and to keep it in the front ranks of that magnificent future?

